# Visualization as irritation: producing knowledge about medieval courts through uncertainty

**DOI:** 10.3389/fdata.2024.1188620

**Published:** 2024-05-10

**Authors:** Silke Schwandt, Christian Wachter

**Affiliations:** Digital History, Department of History, Bielefeld University, Bielefeld, Germany

**Keywords:** uncertainty, knowledge production, visualization, semiotics, theory

## Abstract

Visualizations are ubiquitous in data-driven research, serving as both tools for knowledge production and genuine means of knowledge communication. Despite criticisms targeting the alleged objectivity of visualizations in the digital humanities (DH) and reflections on how they may serve as representations of both scholarly perspective and uncertainty within the data analysis pipeline, there remains a notable scarcity of in-depth theoretical grounding for these assumptions in DH discussions. It is our understanding that only through theoretical foundations such as basic semiotic principles and perspectives on media modality one can fully assess the use and potential of visualizations for innovation in scholarly interpretation. We argue that visualizations have the capacity to “productively irritate” existing scholarly knowledge in a given research field. This does not just mean that visualizations depict patterns in datasets that seem not in line with prior research and thus stimulate deeper examination. Complementarily, “irritation” here consists of visualizations producing uncertainty about their own meaning—yet it is precisely this uncertainty in which the potential for greater insight lies. It stimulates questions about what is depicted and what is not. This turns out to be a valuable resource for scholarly interpretation, and one could argue that visualizing big data is particularly prolific in this sense, because due to their complexity researchers cannot interpret the data without visual representations. However, we argue that “productive irritation” can also happen below the level of big data. We see this potential rooted in the genuinely semiotic and semantic properties of visual media, which studies in multimodality and specifically in the field of *Bildlinguistik* have carved out: a visualization's holistic overview of data patterns is juxtaposed to its semantic vagueness, which gives way to deep interpretations and multiple perspectives on that data. We elucidate this potential using examples from medieval English legal history. Visualizations of data relating to legal functions and social constellations of various people in court offer surprising insights that can lead to new knowledge through “productive irritation.”

## 1 Introduction

Uncertainty has become an essential topic of methodological and theoretical debates on data analysis. Many authors address uncertainty as ingrained in data-driven research while emphasizing the many nuances and types of the phenomenon. For instance, uncertainty may refer to imprecision, error, missing values, and noise (Boukhelifa et al., 2017), the lack of information “due to randomness, such as results by chance” (Bonneau et al., 2014, p. 8), and questions about how to model uncertainty in data. Uncertainty is also involved when researchers decide which tools to use and how to set them up to achieve analytic results. For the digital humanities (DH), more specifically, another dimension is the contingency of data interpretation, which Benito-Santos et al. (2021, p. 2) attribute to “a lack of ground truth” of the involved interpretative perspectives. This becomes particularly apparent in collaborative DH projects, where team members negotiate divergent and often interdisciplinary viewpoints. While these examples certainly are not exhaustive, they nonetheless illustrate the versatile nature of uncertainty and its many context-related synonyms—such as “ambiguity,” “contingency,” “multivocality,” etc.—in DH research. Therefore, taxonomies have been discussed, differentiating, for instance, between aleatoric uncertainty (statistical or stochastic uncertainty of probabilities) and epistemic uncertainty (researchers lacking knowledge about research objects; Fisher, 1999), with the latter manifesting in imprecision, ignorance, credibility, and/or incompleteness (Benito-Santos et al., 2021, p. 4).

At this point, one might question the aptness of such a broad term as “uncertainty,” which itself might appear rather fuzzy. Nevertheless, DH researchers refer a lot to uncertainty, which emphasizes the unquestionable fact that they deal with indefiniteness on many levels of their research practices. This understanding is largely accepted in the DH community, and uncertainty has been embraced by many as an overarching category; publications such as the papers of *Informatics'* Topical Collection on “Uncertainty in Digital Humanities (Theron et al., 2023)” exemplify this tendency. Bonneau et al. (2014, p. 5) state that “uncertainty can arise in all stages of the analysis pipeline, including data acquisition, transformation, sampling, quantization, interpolation, and visualization.” Regarding the first of these stages, data collections stem from specific perspectives and interpretations, as Drucker (2011) emphasized with her concept of “capta” or Lavin (2021) with the term “situated data.” Drucker argued that this fact must be reflected both in the research process and in publications, in order to create transparency and avoid the appearance of positivism. She explicitly demanded that “humanistic visualizations” should highlight this very constructivist dimension (Drucker, 2014, p. 135–192). Gaps in data or any other ambiguity should also be directly visible. One might not know exactly how much and what data is missing (think of archival material lost over the centuries that could not be digitized), but it is crucial to indicate blind spots in the dataset to sensitize users to this uncertainty. Against this backdrop, uncertainty is being discussed as an intrinsic quality of digital research to be made explicit through modeling and transparent communication, to substantiate the rationale of research results. The challenge is to document sources of uncertainty, to make them manageable among project team members, but also comprehensible for readers of publications and a broader audience of science communication.

Visualization is one tool to achieve that goal. Particularly the area of “Visualization for the Digital Humanities” (VIS4DH) has eagerly picked up the challenge of “navigating uncertainty in the digital humanities” (Panagiotidou et al., 2022, p. 641). A common claim within the VIS4DH community is that visualizations should communicate as clearly as possible the various manifestations of uncertainty for respective stages of the analysis pipeline. As Bonneau et al. complain, however, only a few studies follow that imperative. Instead, uncertainty is often omitted (Bonneau et al., 2014, p. 13). In these cases, visual representations tend to become black boxes, undermining any proper declaration of uncertainty as an integral component of data-driven research. Greater consideration of mechanisms of trust-building (when users interact with machines), declarations of analytic provenance, and uncertainty propagation can help to avoid such pitfalls, as Sacha et al. (2016, p. 241–42) argued. This orientation toward the user underscores the need for human-centered visualization designs in visual analytics. More specifically, transparency becomes only possible with visualization designs that are tailored to the respective audience, as the work on visualizations as tools to problematize imprecise, contested, and missing data by Windhager et al. (2019) demonstrated. Focusing on cultural collections, Windhager et al. differentiated between expert and casual users. Both groups have different expectations, previous knowledge, and expertise. This calls for adjusted visualization designs. Furthermore, Conroy et al. (2024, p. 2) reasoned about the formalization of uncertainty “as the quantification of doubt about the measurement result.” In discussing this phenomenon for network analysis, they argued that the objects of this kind of formalization are seldom fixed values. Instead, it is about formalizing probabilities and “ranges of values.” Visualizations might foreground this when displaying visual variables as “uncertainty markers” (p. 7). Conroy et al. also suggested crafting different visualization versions to highlight uncertainty. These versions would then follow “what-if scenarios,” simulating how networks change when specific variables are manipulated or removed (i.e., for network robustness tests; p. 8).

We certainly believe that these means are crucial to navigating uncertainty in DH research. We also believe, however, that the apparently underlying understanding of controlling or at least harnessing uncertainty through transparency is just one side of the coin of conceptualizing uncertainty. Additionally, we would like to discuss uncertainty as a productive force in visual analytics. More precisely, we envision a notion of uncertainty that manifests itself when researchers use visualizations as analytic tools and detect surprising patterns that seem to be at odds with previous knowledge and expectations about a specific topic. In addition to that, uncertainty is at play when a visualization itself generates puzzlement about its meaning and triggers questions about what data is present and what is missing in the visual representation. As we will lay out in more detail in the following sections, such surprising findings *irritate* and allow to critically question how we approach the visualization and, ultimately, data. Therefore, we define *productive irritation* as another dimension of uncertainty and as an epistemological tool to gain new knowledge in DH research. To be sure, visualizations have long been discussed as exploratory devices. However, this concept of open exploration is not exactly the same as emphasizing the positive rupture of productive irritation which has largely remained under-exposed in the methodological and theoretical debates around visualizations. We attribute this epistemic potential to the basic semiotic features of visual media: They have a pictorial and two- or three-dimensional “syntax,” as Staley (2014) put it, which profoundly distinguishes visualizations from texts. As we would like to discuss more closely in the next section, it is above all the holistic overview and interactive manipulation by the user that yield possibilities for productive irritation that textual media do not.

## 2 Revealing clarity through ambiguity: the power of visual representation

Already in 2008, Martyn Jessop wrote about “visualization as a scholarly practice” (Jessop, 2008) and argued for the analysis of such practice as a practice of knowledge production. He rightly stated, that “graphic aids to thinking are not new” but date back to, e.g., Leonardo da Vinci whose notes and scribblings can be described as “sophisticated linkages between thought, images, and text” (Jessop, 2008, p. 281). What is new, in fact, is the way in which visualizations are being used and produced in the humanities since the advent of digital methods, e.g., in the various fields of research often summed up as DH. But what is a visualization? For Jessop (2008, p. 282) it is a method “for creating images, diagrams, or animations to communicate a message.” This message, the character of a visualization as a carrier of information, is key to its use in research.

In the DH, any expression of metrics usually means visualizing quantitative data, although qualitative metrics are also being visualized. As Drucker (2021, p. 86) summarized: “All information visualizations are metrics expressed as graphics.” This has been subject to a vast field of methodological and theoretical debate for a long time and led to the development of an array of tools. We cannot summarize this in full depth here, but the main point at this juncture is that visualizations commonly represent frequencies or other statistical features of data, usually feeding into one or more of the following major characteristics: First, visualizations can be the result of statistical analysis. Second, visualizations can then be used to explore data to gain information that may be part of a research process. This way, visualizations themselves become analytic devices. Interactive and scalable designs make that possible (Ferster, 2013). Third, visualizations can be used as communicative devices to present complex matters that elude proper expression through the linear structure of text (Wachter, 2021b). Another application is the simplification of ideas by giving them a graphical form. In all of these cases, visualizations serve as “meaningful conceptual tools of inquiry and insight in their own right” (Staley, 2014, p. 40).

Except for the purpose of data exploration, the quantitative character of most visualizations creates a seeming facticity that can easily be criticized as utilitarian and positivistic in a way that seems to be rather alien to the humanities (Bubenhofer, 2016, p. 351). Then why do it at all? As one possible answer to that question, we would like to argue that visualization practices in the humanities, especially in history, serve the purpose of scholarly innovation and knowledge production by productive irritation (see above). Historians are used to working with heterogeneous material and creating knowledge through interpretation. And this interpretation is a conscious process. Especially in the analysis of earlier historical periods, the evidence that we rely on in our interpretations can be scarce. Hence, interpretations often integrate knowledge produced by other researchers. Writing about, e.g., the works of St. Augustine in the twenty-first century is highly dependent on knowledge formed over several centuries. This practice of certainty formation follows the famous metaphor of “standing on the shoulders of giants,” but it becomes challenged by quantifying textual properties.

This is because dissecting a text, analyzing tokens rather than words, sentences, and paragraphs, quantifying morphological information and metadata bear data patterns that often bring about new insights and thus academic innovation. That may manifest in quantitative detection of stylistic properties and stylistic evolution of an author, text reuse, or patterns in references to specific persons or places. These patterns and other analytic results then become the raw material for close inspection and interpretation, since historical data gains meaning only when put into adequate historical contexts (Schwandt, 2022). The stylistic evolution of an author's writings, for instance, becomes informative in light of significant changes of the underlying (political, academic, etc.) circumstances, which themselves must be explained by discussing the (cultural, political, social, etc.) developments of that author's environment. Only through this interpretative process do data and information as raw materials transform into historical knowledge. However, this transformation is hardly possible without visualizations. Visual representations serve as gateways to abstract metrics. Knorr Cetina (2001) argued that, from a sociology of science perspective, the fact that research questions and insights are discussed and reflected based on visualizations is in itself an important development in sciences. And while her research concentrates on visualization practices in physics,^1^ it is precisely this new practice of interacting with hitherto unknown or at least not widespread practices in the humanities that promises innovation. Visualization methods in the humanities can be productively irritating in this sense: They render our prior knowledge uncertain and allow us to ask different questions, which, in the end, may lead to new knowledge. While the potentials of visual analytics have been discussed broadly in research on big data visualizations, the critical reflection of visualizations as a research practice is independent of the big data paradigm.

But what are the driving forces for irritation through visualization giving way to deepened insights? In other words, what qualities make the visual format so suited to productive irritation, in contrast to other media formats? There are good reasons to answer this question from a praxeological perspective like Knorr Cetina's. This is because praxeological scrutiny can reveal in-depth the mechanisms of conspicuous patterns stimulating researchers to take a closer look at the represented data, launching a dynamic process of exchanging ideas with colleagues, and pursuing more detailed analysis. At the same time, praxeological approaches make critical interventions to the fact that visualizations are often used in rather unreflected ways, not seizing their potential for visual analytics.^2^ As our own intervention, however, we propose taking a step back and shed some more light on the semiotic foundation for any of such practices: To fully understand how a visualization lets researchers engage with irritating and potentially meaningful patterns, we need to assess the aesthetic properties of that visualization. In this context, “aesthetics” does not refer to the beauty of a visual format. Instead, following the ancient Greek roots of the term, it means the “how of perception” (Schnell, 2001, p. 73) induced by a media product. This understanding is widespread in media studies.

For this kind of analytical and terminological framework, Gunther Kress and Theo van Leeuwen provided important insights. Their work on a “grammar of visual design” (Kress and Leeuwen, 2021) draws special attention to the composition of visual elements into something meaningful as a whole.^3^ Many abstract visualizations are, following this understanding, “analytical structures.” They represent individual elements with emphasis on their relationship to the entire composition (p. 92–101). Kress and van Leeuwen call this “meronymical (‘part of') relations” (p. 76). They define visual formats such as network graphs with nodes and edges as their key elements more specifically as “connected analytical structures,” since they represent both: elements with their connections to each other and to the whole.

Other approaches from media studies, semiotics, *Bildwissenschaft*, art history, or other image-related research areas also illuminate visualizations and their semiotic, communicative, and esthetic effects. Multimodality Studies (Norris, 2016; Seizov and Wildfeuer, 2019), significantly shaped by their pioneers Kress and van Leeuwen, are particularly informative for our discussion of visualization and productive irritation because they combine two aspects: Multimodality Studies (1) analytically assess image components and configurations, and they (2) do not limit themselves to focusing on visual phenomena, but highlight contrasts and interplays between images, language, and other modalities. “Modality,” in this context, refers to a particular sensual experience caused by a specific channel of communication. For instance, a network graph evokes a holistic overview of abstractly depicted and interlinked data points or information chunks because of the graph's pictorial modality. A textual description of this constellation would be much more precise in terms of articulating relationships between nodes and edges. Still, we would read that extensive description successively, in contrast to the holistic impression of the visualization. This is due to the modality of (written) language. When we find textual labels in the visualization, co-texts beneath it, or any other type of image-text-combination, we encounter an interplay of both modalities. “Interplay” is the operative word here, because the modality effects do not merely add up. Instead, the textual components may clarify the visual representation, and, vice versa, the visualization may render the written account more comprehensible or highlight structural information that does not appear in the text. Hence, there is a convergence of both modalities at play, not the sum of its elements. In Multimodality Studies, this is referred to as “semantic multiplication” (Lim Fei, 2004, p. 239). As a vein of multimodality research, *Bildlinguistik* has a special focus on converging pictorial and linguistic properties (Diekmannshenke et al., 2011). As shown in Table 1, Hartmut Stöckl presented an overview of these fundamental properties that already hints at the fact that the semantic vagueness of visualizations (as images) is counter-weighed by their clear way of presentation.

**Table 1:** A comparison of semiotic modes—picture vs. language (reconstruction of Stöckl's original table; Stöckl, 2009, p. 8).

	**Picture**	**Language**
1. Semiotics (sign system)	Continuous “flow” of signs	Discrete, distinct signs
	Integrative grammar (weak)	Combinatorial grammar (strong)
	Spatial configurations	Linear units (syntagmatic)
	Iconic (close to perception)	Arbitrary (removed from perception)
2. Perception/cognition (understanding)	Simultaneous—holistic perception	Step-by-step
	Quick	Slow (comparatively)
	Strong in impact and memory	Weaker in impact and memory
	Directly tied to emotions	No direct tie to emotions
3. Semantics (meaning potential)	Surplus of “free-floating” meaning (semantically dense)	“Anchored” meaning (semantically scarce)
	Vague and under-determined	Precise and determined (tendency)
	Limited semantic range, e.g., negation, modality, abstract reference, illocutions, and linking of utterances	Unlimited semantic range
4. Pragmatics (communicative functions)	Presentation of objects rich in perceptible properties	Narrating actions/events in time
	Indicating relational position of objects in space	Explaining logical relationships between entities
	Emotional appeals	All illocutions and speech events

Courtesy of Hartmut Stöckl.

Stöckl's multi-level analytical differentiation between image and language outlines qualities that one might intuitively attribute to visualizations. However, some combinations of modality properties are less intuitively explained: It is remarkable that, on the one hand, the *integrative grammar* of images creates an overview of spatially arranged and feature-rich objects, thus mapping constellations with high accuracy.^4^ On the other hand, images are vague in the sense that viewers can extract more meaning from them—also: easily project meaning into them—than one would sensibly do with well-formulated linguistic sentences. Thus, semiotic concreteness goes hand in hand with semantic richness and vagueness, which reveals a certain tension.

And this is exactly what working with data visualizations as tools of knowledge creation often means: Patterns become visible, yet their exact meaning must be explored by zooming into the corresponding areas. Put linguistically, visualizations provide complexes of propositions (cf. Große, 2011, p. 118)—individual statements or propositions on the data exist in abundance but only implicitly due to the vagueness of visual representation. They must be made explicit in assessing the visualization and interpreting the visualized elements, their relationships with each other, and, following Kress and van Leeuwen, with the overall depiction (cf. Haas and Wachter, 2022). Only then we can formulate specific statements about the visualized correlations and patterns.

This approach to data visualizations as analytical tools is complemented by the communication function. When researchers work together or when they publish their findings, visualizations serve as presentation tools to clarify which data points and patterns are under discussion. Visualizations are often crafted as interactive devices, open to manipulation by various users to enable contingent interpretation. For instance, the tool *Stereoscope* lets literary scholars visualize their own manual annotations of text material (3DH, 2016–2018). This “hermeneutic visualization” records and communicates qualitative statements about those texts. Rabea Kleymann and Jan-Erik Stange called this visual practice the “use of computer-supported, interactive, visual representations of text annotations to manipulate, reconfigure and explore them in order to create visual interpretations that can be used as arguments and allow a critical reflection of the hermeneutic process in light of a research question” (Kleymann and Stange, 2021). This multivocal approach toward data directly follows a critical imperative: Multivocality itself is meant to be visible and explorable in the visual representation. How data is to be interpreted becomes a task of uncertainty in the sense that there are multiple, contingent ways of interpretation. And it is the semantic density and vagueness of visualizations as pictorial formats that support this mission because they open up a space of possibilities for exploration, instead of determining rigid interpretation guidelines.

## 3 Producing uncertainty in historical material/in history: productive irritation

Visualization as a method of data exploration is especially useful in the context of the analysis of big data sets because it relies on statistical and probabilistic calculations. Unfortunately, most medieval history projects do not deal with large bodies of data which can be described as big data in this sense. Drucker (2020, p. 111–112) has prominently argued that this is the biggest challenge for humanists when dealing with formal modeling practices which are key for the creation of visualization: “Not only are the materials of the humanists unable to be represented adequately by the points, dots, bars, and lines of conventional charts, even at the level of abstraction, but more importantly, the processes of doing humanistic work [...] are not accommodated by the graphical means designed for empirical and statistical sciences characterized by discrete components and disambiguated features.” And while it is true that humanistic interpretation often resists formalization, the challenge itself can be productive because of the change in perspective. This will be one of the main points with the following examples taken from a project on medieval English legal practices. For history, the body of court rolls from the National Archives in London would qualify as a body of big data since there are thousands of protocols (so-called court rolls) from about 1180 up until the nineteenth century that can be accessed in the archive. Hence, quantitative and computational methods seem to be well-suited to analyze these sources, and the amount of the resulting data is big enough to use visualization techniques to look for patterns and insights present in the material which might not be detectable by reading alone. Leaving the sphere of reading in a sequential manner, visualizations produce uncertainty about previous assumptions concerning the material. But science is about conquering the unknown, there is no scientific innovation without uncertainty. Using data visualizations as part of the interpretation process produces uncertainty and provides a different kind of evidence that provokes new interpretations which can productively irritate previous knowledge (Schwandt, 2018). Allowing for the production of uncertainty opens up new spaces of interpretation.

Digital methods of data exploration and visualization allow for productive irritation by providing new perspectives on text, for example. Reading written primary sources in history leads to certain interpretations that rest on practices of following sequential argumentations and linear narratives. Reading data visualizations irritates this practice and asks for a new visual literacy in order to interpret diagrammatic relations. In this sense, visualizations produce uncertainty for the interpreter—but visualizations also have to deal with uncertainty in the material and data itself. Therefore, Fafinski and Piotrowski (2020) called for more attention to the process of modeling uncertainty in historiography as well as in history. As we will show in this article, modeling uncertainty can be a mode of productive irritation.

Implementing digital visualization practices in historical research is a complex task that starts with matching data and tools with historical research questions. Quantification is not alien to historical research and has been used frequently in social and economic history. With the linguistic turn, quantification has also entered the field of conceptual history and historical semantics focusing, e.g., on the analysis of word frequencies and co-occurrences (Müller and Schmieder, 2016). But in order for quantification to offer interesting results, it is necessary to carefully build corpora as data for visualizations. Most data science methods use visualizations to detect patterns in unstructured large bodies of data, e.g., survey data or digitally produced data like social media data. For historical projects, this data needs to be created, often in a process of retro-digitization following different steps of Natural Language Processing (NLP; Jentsch and Porada, 2020). The annotation of text material with linguistic information and meta data allows for multi-level queries regarding the contexts of word use to analyze concepts and meaning in discourse and to identify, e.g., the role and function of so-called named entities like persons, locations or institutions as they are mentioned in texts. But which digital or computational methods match the social history questions one would ask in the analysis of the source material? Designing projects in DH is an interdisciplinary endeavor, producing uncertainty on two levels: “historians are uncertain how they as historians should use digital methods, and computational experts are uncertain how digital methods should work with historical datasets” (Kemman, 2021, p. 3). In practice, analyzing visualizations as part of the research process is a task of navigating uncertainty to achieve innovation.

### 3.1 Case study: analyzing medieval court protocols

In the following examples, using digital methods, we will interrogate the court rolls about the practices, roles and functions during court sessions, and the relationships between litigants present. The English legal system in the Middle Ages was shaped by a plurality of courts with different regional jurisdictions ranging from village courts to county courts and all the way up to the King's Bench in Westminster. All of them adhered to the Common Law, guaranteed to everyone by the Magna Carta which was first issued in 1215. One peculiar institution were the so-called Justices in Eyre, who were officials in the service of the king and visited localities to dispense justice as members of the royal household and later as members of the King's Bench (Baker, 2002, p. 16). The Eyre meetings were not the highest court in England, but the justices had the widest jurisdiction and developed the greatest physical presence, as one could experience the royal court on the spot through their visitations. DeWindt (1981, p. 108–111) estimated for the Huntingdonshire Eyre of 1286 that up to 2,000 people were actively involved in the proceedings, either as plaintiffs, defendants, or advocates. The Eyre rolls contain the documentation of the cases heard by the judges, including judgments and the names of the people involved.^5^ The following examples use data from two Eyre court sessions in 1248 in Berkshire and 1286 in Huntingdonshire which have been digitized manually.^6^ Using different methods from the field of NLP, the textual material was enriched with annotations, e.g., Part-of-Speech-Tagging, and Named Entity Recognition, which entail information about words and tokens as quantifiable information.

From a medieval history perspective, the material has been analyzed as documentation of the process of institutionalization of the Common Law (Hudson, 1996), or as basis of the development and formalization of legal procedure, e.g., in the case of specific administrative developments (Caenegem, 1972). The following examples concentrate more on the analysis of the social structures and practices involved in legal practice: What do people do in court? Who is involved? For this reason, the visualizations will try to explore the wording of particular actions in court and the social relations of the people present at court. Furthermore, the examples and the underlying hypotheses are designed in an explorative and simplistic manner to illustrate the productive character of working with visualizations in the research process.

### 3.2 Practices, roles, and functions at court

English legal practice is a social practice by means of which social status, relationships and hierarchies on the one hand, and (economic) resources on the other hand, are negotiated. All these aspects do not only concern the actors in court, e.g., plaintiffs and defendants, but also other staff, people who were involved in the court session: judges, jury members, and attorneys. Many of these actors are not professional members of a court. Courts convene and are made up of people for whom court service was only one of many (often vassal) duties. That is: at the moment of the court, at the moment of the trial, a group forms for a specific purpose. Who is involved in these group formations? From which social groups do the actors come and in what ways do they interact with each other? What claims are formulated, who sends the judges, from which contexts do the involved jury members come?

**Figure 1:**
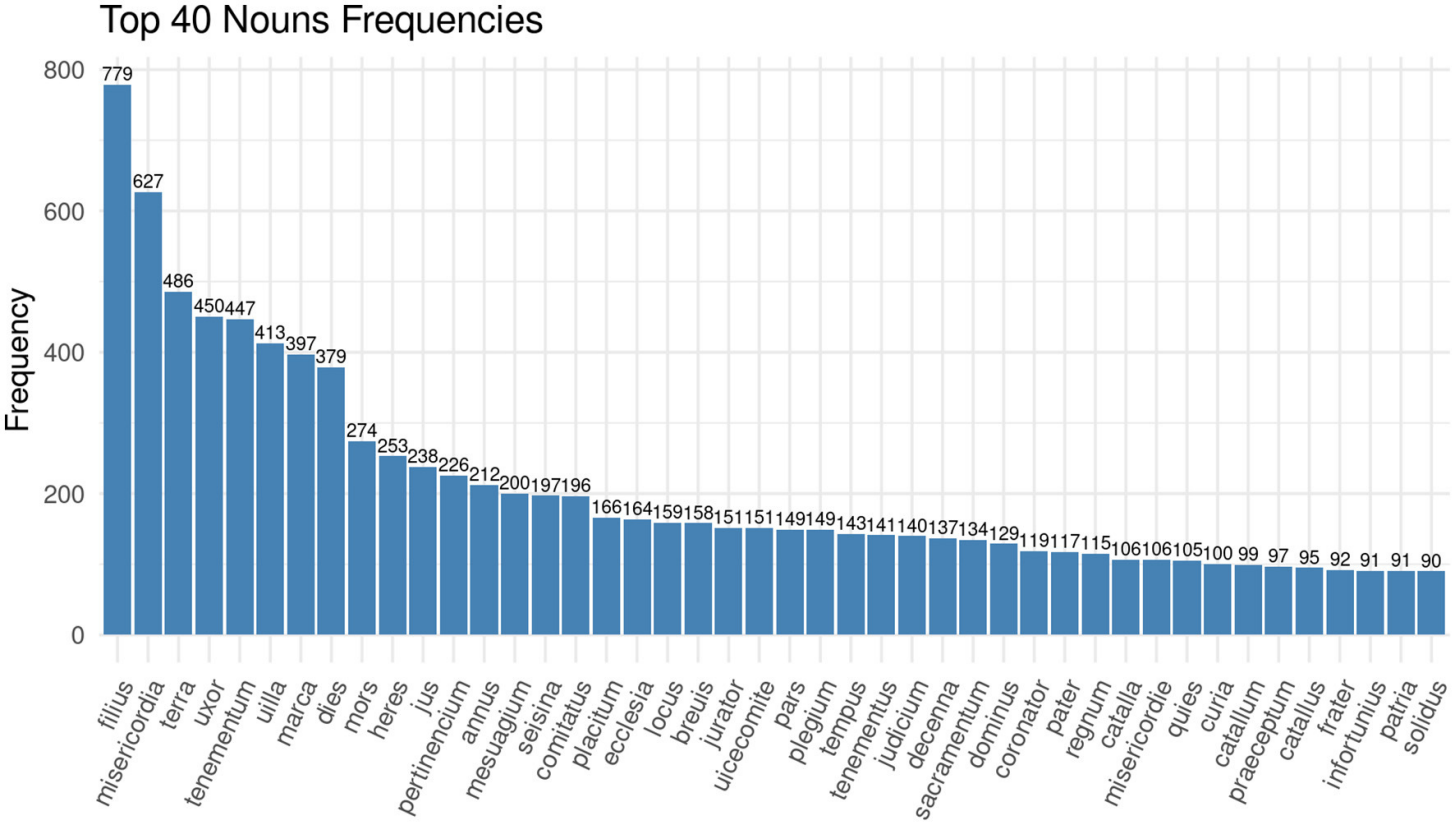
Bar chart showing the 40 most frequent nouns in the corpus of the Huntingdonshire Eyre rolls of 1286.

It is particularly interesting for the analysis of medieval legal practices to identify the roles and functions that needed to be fulfilled during the court sessions since they represent the interaction of the participants within a specific social setting. For exploration purposes, we performed a keyword-in-context search looking for words, specifically nouns, which would convey the different roles of, e.g., judge, litigant or juror. Against our expectations, none of those words are part of the 40 most frequent nouns in the documentation of the Huntingdonshire Eyre of 1286 (see Figure 1). In fact, *jurator* (English: juror) appears 151 times on place 21 in the top 40 list, *justiciarius* (English: *justiciar*) only appears five times while *filius* (English: son) appears 779 times as the most frequent noun in the corpus.^7^

**Figure 2:**
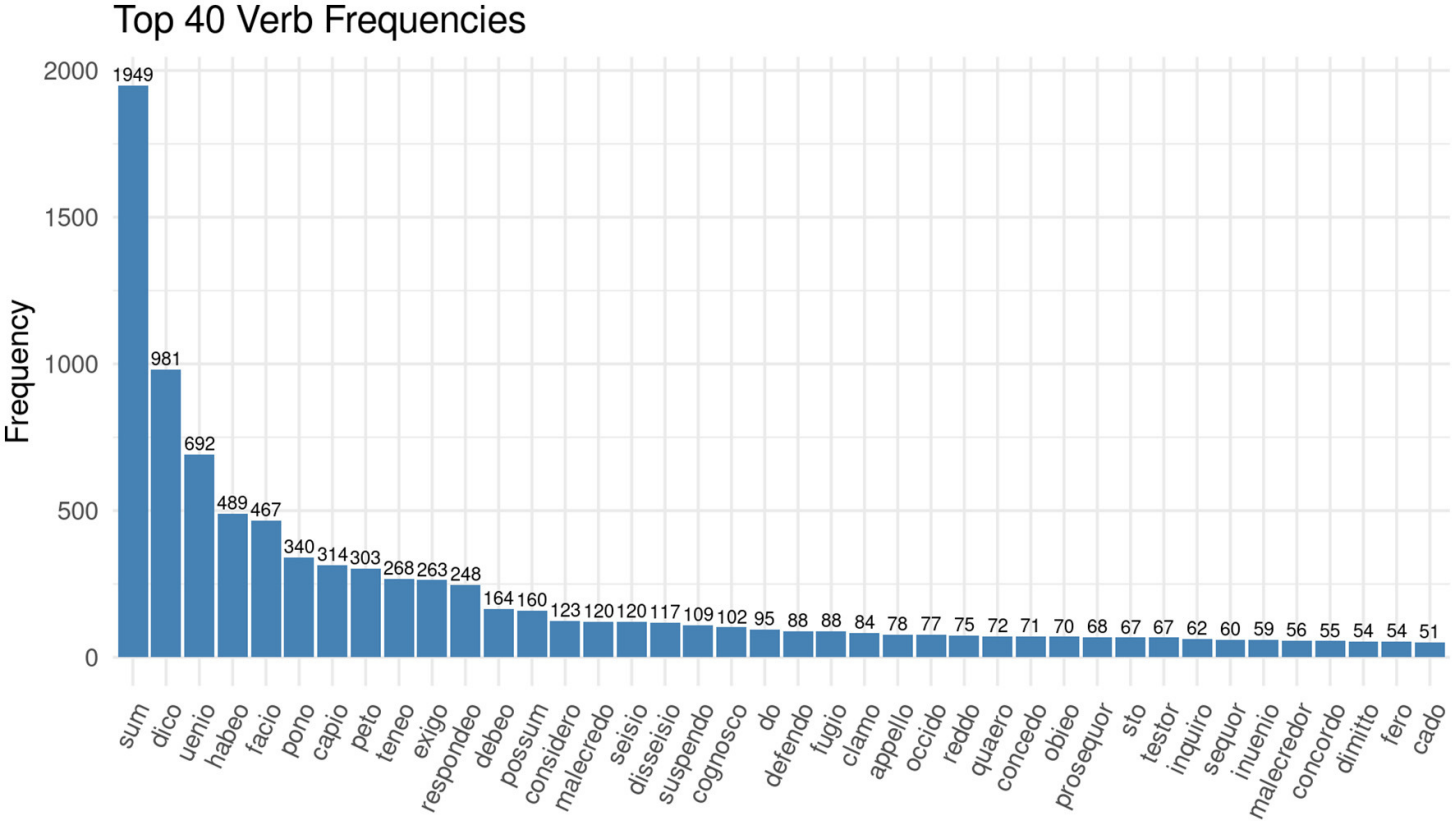
Bar chart showing the 40 most frequent verbs in the corpus of the Huntingdonshire Eyre Rolls of 1286.

Among the most frequent nouns are instead terms which denote the outcome of a case, such as *misericordia* (English: amercements) or *judicium* (English: judgment), and terms which denote familial relations, such as *filius* (English: son) and *uxor* (English: wife). We will have a look at the personal relationships present in the documents later. The data exploration at this stage raises questions in the way that we described as productive irritation: if there are so few nouns describing the functions at court, how are the proceedings framed linguistically? If we look at the most frequent verbs in the corpus of the Huntingdonshire Eyre, the answer becomes clear (see Figure 2).

What people did at medieval courts is not predominantly denoted by nouns but rather by verb forms. Observing the frequencies of the verbs, and disregarding *sum* (*esse*, English: *to be*), because it is always the most frequent verb, it becomes clear, that everything revolves around *dico* (*dicere*, English: *to say*) and *uenio* (*venire*, English: *to come*).

**Figure 3:**
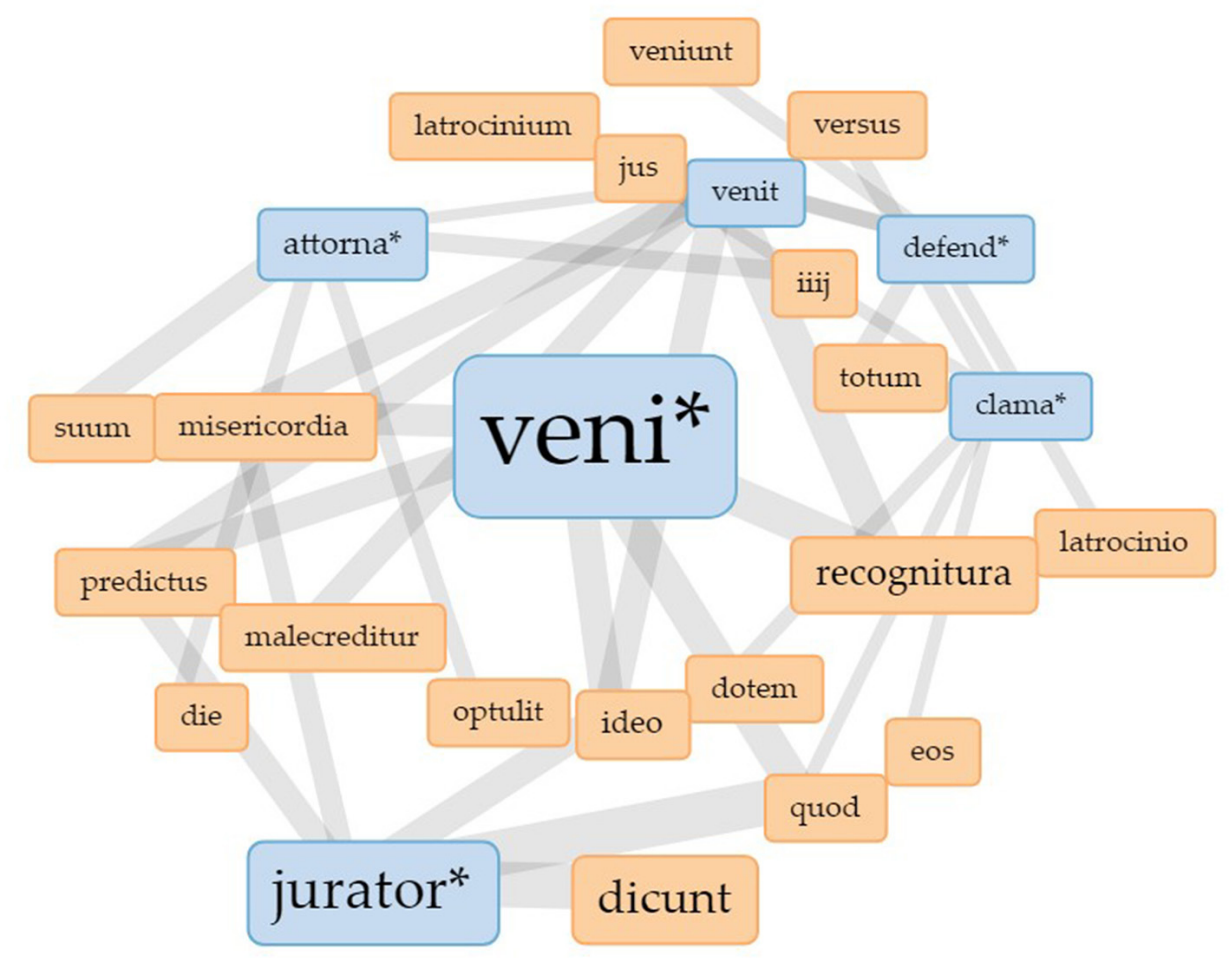
Collocates graph for “veni^*^” in the Eyre roll of the Berkshire Eyre of 1248. Visualization realized with Voyant Tools.

To explore this observation further, digital visualization methods provide even more possibilities. Figure 3 shows a Collocates Graph (Sinclair and Rockwell, 2023a)^8^ for the Latin verb *venire* (English: *to come*; here shown in the truncated form “veni^*^” to include all possible conjugated forms of the verb). The data base for the visualization are the court protocols of the Berkshire Eyre of 1248 as they were edited by Michael Clanchy. These protocols contain information on the cases heard in front of the royal judges during the Eyre as well as on the people involved and on their actions during the court sessions. The edition was transformed into txt-files and cleaned of headlines, footnotes and other marginals present in the modern text. The material was then uploaded to *Voyant Tools* to create the visualization. The terms in the blue boxes are manually selected keywords. *Venire* is one of the most frequent words used in the Eyre rolls since it first and foremost denotes the activity of “going to court.” In order to be able to relate this importance of *venire* to other activities in court, the keywords chosen for this visualization all denote central activities: to judge (jurator^*^), to defend (defend^*^), to speak for someone else (attorna^*^) and to call out or to speak loudly (clama^*^). The terms in the maroon boxes are the collocates of the keywords, i.e., the words that appear within a 10-word context, five words to the left and five words to the right of the keyword. The lines connecting the nodes in the graph show that some of the keywords share the same collocates.^9^ The centrality of *venire* in the graph shows its relevance to court procedure: *venire* is the central activity.

While these observations already build on the uncertainty produced by the unfulfilled expectation to find more nouns denoting functions, the visualization itself triggers new uncertainty as to how to read it properly. The network visualization in Figure 3 is a force directed network graph designed to be easily readable. The algorithm assigns forces to the nodes to arrive at edges of comparable lengths which cross as seldom as possible. And although we would like to assign meaning to the layout and the position of the nodes, the algorithm does not provide meaningful information in this way. While the position of the nodes, thus, does not necessarily carry meaning, the size of the nodes and the width of the edges do. The size of the nodes represents the frequency of the depicted terms while the width of the edges represents the strength of the connection, i.e., how often the terms connected with a line co-occur. The wider the line the higher is the frequency of co-occurrence. This provides insight into the word structure of the Eyre rolls (here, of the roll for the Berkshire Eyre in 1248) and the way that the different functions in court, represented by the verb forms, are linked to each other. In this sense, the network does not only depict the relationship of words, but also of practices. The graph shows the various connections of the terms but it hides those terms that do not co-occur with the keywords we started with.

What we do learn from this graph is what people do when they come to court: they talk, they judge, they defend. So far, this was to be expected. Apart from the observation that nouns are less frequent than verb forms which tells us something about the overall linguistic structure of medieval Latin, this network shows the relationship of practices at court. Talking, judging, and defending are dependent on *venire*, which means that these practice bundles (Schatzki, 2019) are dependent on the actual physical presence at court. It would be interesting to see next what happens when we take *venire* as the central activity out of the picture.

**Figure 4:**
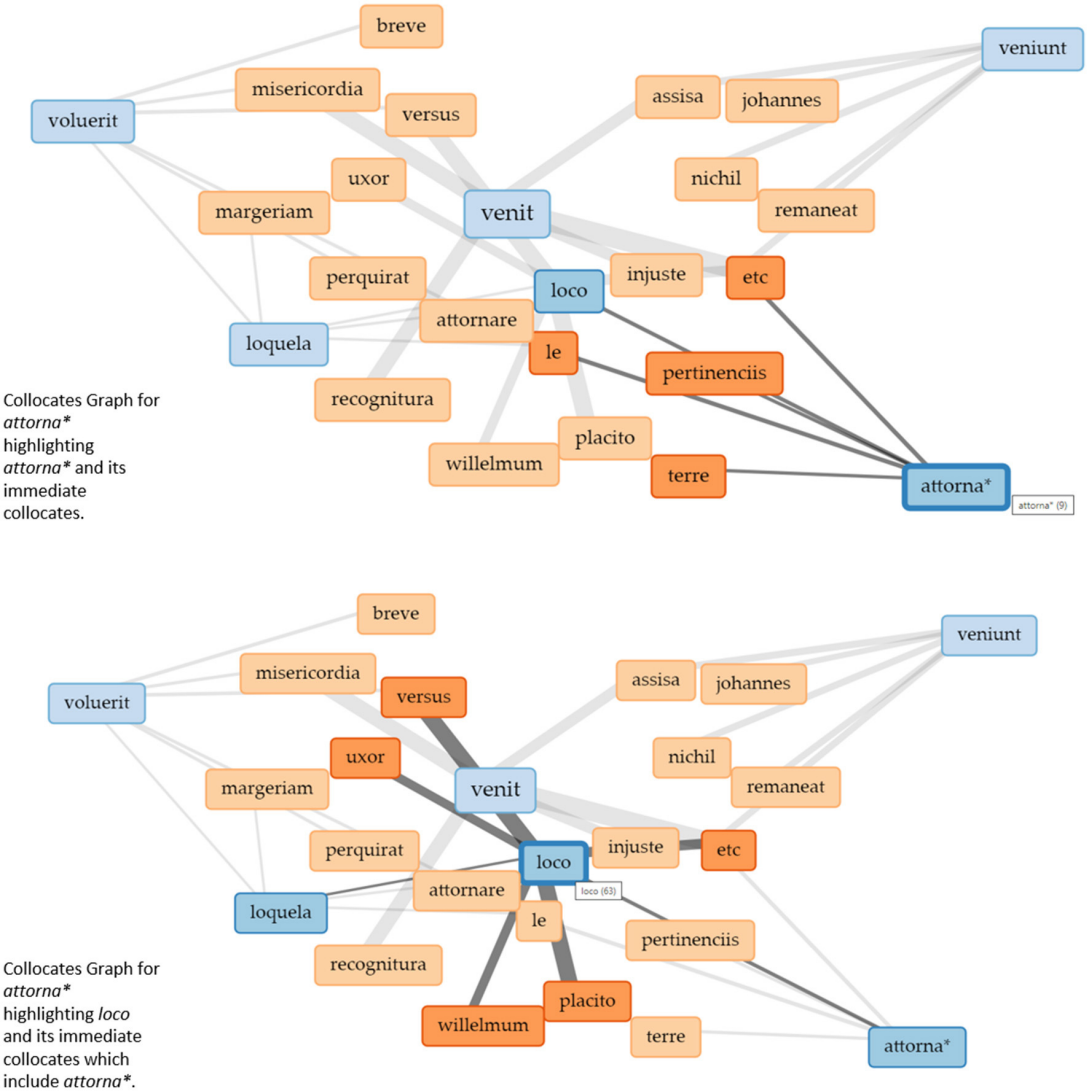
Collocates graphs for “attorna^*^” in the Eyre roll of the Berkshire Eyre of 1248. Visualizations realized with Voyant Tools.

Figure 4 shows a Collocates Graph with *attornare* (truncated attorna^*^) as the chosen keyword. The context for the collocates was set to 15 words to the left and 15 words to the right because *attornare* is much less frequently used in the corpus than *venire*.^10^ The terms that also appear in the blue boxes here are terms that are connected to attorna^*^ but are themselves more frequent in the corpus. They are then shown with their own collocates.

What becomes apparent is that veni^*^ again is the central activity because it governs other practices—even if it is not one of the preselected keywords.

The visualization of collocates in the form of a network helps to learn about the social practices and their relations present within the corpus that we would not have been able to detect simply by reading the documents. With text (and specifically court rolls) being almost the sole source of data about the medieval legal system, understanding verbs as practices and using visualizations to learn about their relations produces new forms of knowledge that will be used to read texts differently. In this sense, again, we use visualizations to produce uncertainty through irritation. But similar to interpreting text, the interpretation of the collocates graph picks up on some of the data represented without knowing the whole picture. The nodes are predetermined by a search for specific keywords, which connects to other concepts of uncertainty discussed above since some of the data remains hidden. In this way, the visualization retains vagueness and ambiguity which are necessary for interpretation practices in the humanities (Piotrowski, 2019), while using visualizations as part of the interpretation process at all produces productive uncertainty.

### 3.3 Relationships between litigants

Another way to understand the proceedings in court as part of a social practice is to look at the different relationships between the litigants involved. John Hudson attributes a key function to the private courts of the feudal lords in the regulation of personal relationships in addition to the administration of land resources (Hudson, 1996). With the start of the twelfth century, the private courts attained a formalized position alongside the regional courts and were primarily concerned with lawsuits over land ownership. All parties were bound to each other by one fief or tenancy or another, and so peers judged and sued each other. In this way, a sensitive web of personal and economic, social and hierarchical relationships was maintained, into which outsiders could only be integrated with difficulty. A closed group was constituted that mutually assured each other of their status. Is this visible in the sources? What kind of influence does the fact that the Justices in Eyre were outsiders to these local groups have on them?

#### 3.3.1 Personal relationships

**Figure 5:**
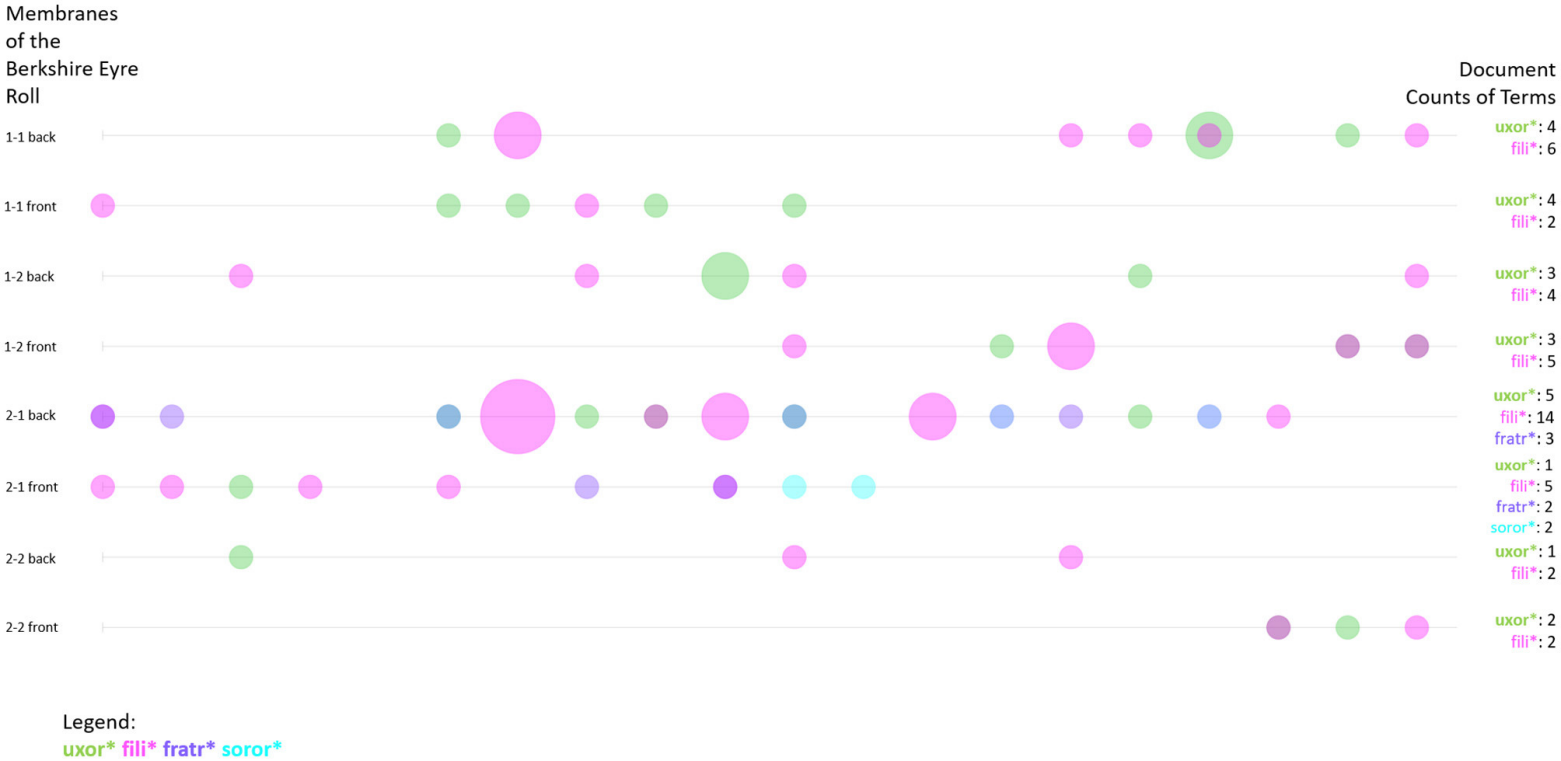
Bubblelines visualization showing the frequency and distribution of the terms uxor^*^, fili^*^, fratr^*^, and soror^*^ in the Eyre roll of the Berkshire Eyre of 1248. Visualization realized with Voyant Tools.

In Figure 1 we have already seen that some of the most frequent terms in the Huntingdonshire Eyre Rolls are *filius* and *uxor*. This demonstrates the importance of familial relations in court—also as a means to identify individuals, since their relation to a specific man as son or woman as wife is what makes them recognizable. In addition to the frequency of words and word combinations, some digital tools allow us to visualize the distribution of these frequencies across the text. The Bubblelines plot in Figure 5 shows the distribution of the word forms of *uxor, filia* (English: *daughter*) and *filius, frater* (English: *brother*) as well as *soror* (English: *sister*). Again, all forms have been truncated to account for all possible word forms (Sinclair and Rockwell, 2023b). Each line represents a document—in the case of the Eyre rolls, this is a membrane, i.e., a piece of parchment that is sewn to other parchments in order to form a roll. Also, the document is divided into 20 segments of equal size. The position of the circle shows the distribution of the terms within the document and its size represents the frequency of a term within the respective segment. In Figure 5 the sizes are all similar, the smallest representing a frequency of 1, the medium size representing a frequency of 2, and the biggest representing a frequency of 4. The document frequencies of the terms are also given in the graph.

A peer group as the one described by John Hudson is usually organized in a familial way in the Middle Ages. Especially women were often only able to act in court as a family member dependent on a male relative like a husband, brother, or father (Seabourne, 2021). This only changed if they were themselves the victim of a crime or if they had attained the legal status of widow which granted a women different rights—also in the terms of land possession (Biancalana, 1988). Therefore, it would be interesting to see how the different terms are distributed throughout the corpus in order to evaluate if and when women (wives, daughters, and sisters) would act independently from men.

Apparently, the terms denoting familial first-grade relations are unevenly distributed. *Frater* and *soror* are not only much rarer in the corpus as a whole, they also appear more isolated in the distribution compared to *uxor* and *filius* or *filia*. Brother and sister do not seem to appear in court together—at least not in the present corpus. However, their position in the immediate vicinity of an *uxor* or *filius* bubble again shows the familial logic of the personal relationships represented. As to the question of uncertainty, the visualization again blurs much of the data in order to highlight specific features. It allows us to focus on specific entities and use them to understand the structure of the groups present at court. The frequent usage of terms of familial relations depicts a group of people who know about those relations and deem them important. An interesting next step would be to look at the combination of relational terms and names.

Table 2 shows the 10 most common n-grams involving one of the familial terms in question. The most common combination is either with the name of a male person (Johannes, Ricardus, Robertus, or Willelmus) or with a form of the possessive pronoun *ejus*. Here, the genitive form in the n-grams marks a clear hierarchical relationship of belonging (to a person or a household). The third most frequent combination is with *heres* (English: *heir*). This also mirrors the fact that *heres* was one of the most frequent terms in Figure 1. Familial relations are therefore relations of hierarchy, of belonging, and of possession or succession.

**Table 2:** Table showing the 10 most common n-grams of minimum 2, maximum 5 word length involving uxor^*^ (wife), fili^*^ (son or daughter; the table shows only filius=son), fratr^*^ (brother), or soror^*^ (sister) in the corpus of the Berkshire Eyre of 1248.

**Term**	Uxor^*^	Fili^*^	Fratr^*^	Soror^*^
Absolute corpus frequency	435	750	58	38
N-grams (min 2-max 5 words)	Uxorem ejus	Filio et	Fratri et	Soror predicti
	Uxor ejus	Filius johannis	Fratri et heredi	Soror ipsius
	Uxorem ejus de	Filio et heredi	Fratri et heredi, et	Sororem ejus
	Uxore ejus	Filius willelmi	Fratri et heredi, et de	Soror predicti simonis
	Uxorem ejus de placito	Filius roberti	Fratrem ejus	Sorori et
	Uxor willelmi	Filio et heredi, et	Fratrem ejus, ricardum	Sorori et heredi
	Uxor roberti	Filius ricardi	Fratrem et	Sororis ipsius
	Uxor ricardi	Filium roberti	Fratrem predicti	Soror predicte
	Uxor roberti de	Filium willelmi	Fratri et heredi, et quod	Soror predicte margerie
	Uxor thome	Filius henrici	Fratris sui	

What this example shows is that the exploration of textual data is an iterative process which involves many different steps to arrive at an interpretation. Not one visualization shows it all. What is also important is that visualizations like the one presented in our examples are meant to be explorative. They help to make sense of the data through pattern recognition on the one hand, and make it necessary to go back to the textual data and the documents themselves to arrive at an interpretation. As part of the research process, visualizations produce uncertainty and irritation which guides us to zoom into the documents themselves to understand the social constellations present at court. Apparently, familial relations can be part of the court proceedings in at least two ways: they serve as identifiers and denote hereditary rights.^11^

#### 3.3.2 Social groups and hierarchies

Hudson's argument about peer groups or familial groups also implies that the groups at court are socially coherent in the sense that they consist of people from the same social strata. This assumption would also fit to the structure of the regionally organized and locally executed jurisdiction in the years of the early common law. While the village courts served mostly a closed group of locals, the Eyres came to the localities but represented direct access to royal justice. In this context, social status became something that was negotiable to a certain extent. How would that be researchable? The cases themselves do not discuss social status, but they were also not only about judgment.

The documentation of the cases of the Huntingdonshire Eyre in 1286 shows that of the 705 cases that were heard in Huntingdon before the Justices in Eyre about half, 356, were civil actions, 191 out of 356 being “civil actions brought by writ,” i.e., civil actions dealing with land ownership (DeWindt, 1981, p. 15). Of these 191 cases only just over a third were pursued to judgment (DeWindt, 1981, p. 35).^12^ Such figures suggest that in many cases it was less about substantive decisions and more about clarifying social and hierarchical relationships between litigants at court. Anne Reiber DeWindt has compiled a biographical register for the Huntingdonshire Eyre Roll, in which she has recorded almost all the people named in the documents. Individuals were identified by last name and then assigned to familial groups that had certain rights, privileges and obligations to landowners, the king or the village community (DeWindt, 1981, p. 66). According to this social profile, the identified people were divided into four categories: knights, regional landholders, villagers, and clerics. The register makes it possible to examine the interaction between people in analogy to the levels of social hierarchy. Here, persons are associated with cases in which they participated. Collecting this data, we created a visualization of cases and participants in order to see the social structures as combinations of members of different social groups.

**Figure 6:**
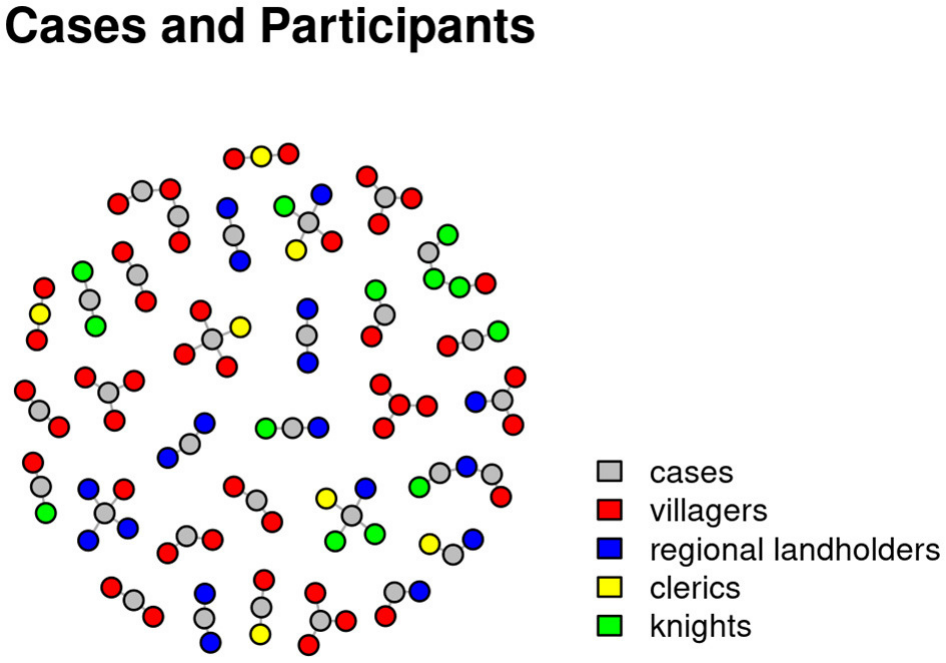
Network visualization showing the people involved in single cases in the Eyre roll of the Huntingdonshire Eyre of 1286. Nodes represent persons belonging to either one of four social categories: knights (depicted in green), regional landowners (depicted in blue), villagers (depicted in red), and members of the clergy (depicted in yellow).

The labeled nodes in the network graph in Figure 6 stand for cases while the colored nodes without labels represent persons involved in the cases. The people were present in court for example as a litigant, or were involved in one of the cases as testator in a case concerning the inheritance of a piece of land. The color coding of the nodes depicts the persons belonging to the four social categories given by DeWindt: red for villagers, blue for regional landowners, green for knights and yellow for members of the clergy. To make the visualization more readable, it shows the names and people involved in one session of the Eyre court and not all sessions of the year 1286.

What we can see here are several small network graphs that show not only the combination of people but also the cases (represented by the gray nodes) that are connected through the participants involved. In this way, court sessions form new, often overarching communities of practice—and here productive irritation comes into play: what does that mean and how does it work?

Again, the data is incomplete, but the uncertainty dimension that we want to address does not come from this incompleteness. We want to address the uncertainty of the interpretation. The space of interpretation opens toward a more dynamic picture of the society in Huntingdonshire at the end of the thirteenth century—at least of the community that took part in the session of the Eyre in 1286. Maybe a network graph for a session of the general county court or a village court would look differently. The Eyre allows for more people to participate in the same court. In his book on the county courts, Robert C. Palmer describes the choice of court as a complex matter, which above all required considerable knowledge of the jurisdiction of courts, the system of tenure and the legal strategies of speech and counter-speech (pleas).^13^ If one assumes that this knowledge was also available to the lower strata of the population, as the surviving cases in the Huntingdonshire Eyre Rolls and comparable documents suggest, then one can also conclude that the actors in court chose quite consciously between different courts and jurisdictions.

Therefore, the graph produces irritation and uncertainty about different assumptions. The most obvious is this: people did not only sue other people from the same social strata. This demonstrates the extent to which the Eyre became an arena of social interaction transcending social categories. The graph suggests that there were multiple connections between the litigants which surpassed the dominant structure of society.

All the examples in this section demonstrated the potential of visualizations as a research instrument to produce productive irritation through uncertainty.

## 4 Discussion: productive irritation and communication of uncertainty through visualizations

Uncertainty plays a significant role in utilizing visualizations for inquiry and communication. As the examples from medieval English legal history have shown, visualizations question previous knowledge and thus draw attention to connections that were previously barely visible. This “productive irritation” of our knowledge is based on constellations and patterns that are themselves characterized by uncertainty: their appearance may look striking to us, but at first glance, we do not know exactly what they mean. By asking what information a visualization depicts and what information it disguises, uncertainty is also rooted in the visual representation itself. Instead of seeing this as a shortcoming, we argued for this semantic uncertainty as a valuable resource for scholarly exploration and interpretations. Interpretations can be diverse and thus are marked by contingency. However, this does not represent inaccuracy or weakness. Rather, it is a multivocality that enriches research discourse, hence improving our knowledge.^14^

As we have explained, this added value of visualizations is due to their semiotic qualities as media. Multimodality research and particularly *Bildlinguistik* provides us with an analytical view to uncover that precisely. Here, it is emphasized that pictoriality leads to a holistic iconic overview, unlike spoken or written language. Information chunks and their relationships to each other and to the big picture are visible at one glance. This concreteness is contrasted by semantic vagueness, as the meaning of such visualized constellations is not firmly anchored in the visual representation. Rather, meaning is implicitly present in the form of propositional complexes and must be explicated by the researcher's interpretations. The visualization itself remains vague or ambiguous and, in that sense, uncertain.

What does this mean for tool development and application in digital humanities? On the one hand, the format and interaction potential of visualizations must be closely tailored to the fundamental research interest, even if we do not know exactly what there is to explore in the visualizations. In the discussed case of medieval English legal history, person networks and constellations of practices are at the center of attention. New findings come about against the background of this epistemic context. Both data preparation and visualization formats must be designed to meet this context. This speaks against a certain “independence” of visualization tools to convey information on data “as such” for “any” kind of exploration. It also addresses questions of scale, since not all historical data sets are big data collections.

Hence, visualizations open up new possibilities for the communication of cultural-historical data. And while the field is currently developing into various directions, the use of visualizations as a research practice for small-scale historical data sets still needs more theoretical reflection. As we have argued in this article, knowledge production is more often than not a business of uncertainty and asks for controllable corpora to establish use cases. By engaging with concepts of vagueness, ambiguity and irritation we can suggest practices of navigating uncertainty in historical research through visualization. For digital humanities research, this offers the potential to break away from linearly oriented narratives (Wachter, 2021a), as supposedly “certain” narratives, and emphasize greater diversity and more complexity of perspectives, as forms of productive uncertainty.

## Data availability statement

The raw data supporting the conclusions of this article will be made available by the authors, without undue reservation.

## Author contributions

All authors listed have made a substantial, direct, and intellectual contribution to the work and approved it for publication.

## Footnotes

^1^Karin Knorr Cetina developed her praxeological perspective for collaborative work in physics. She observed research group members discussing their work with the help of visual media. Knorr Cetina coined the term “Viskurse” (“viscourses”) for that kind of communicative practice (Knorr Cetina, 2001).

^2^Bubenhofer (2018) made this case for Visual Linguistics. He contrasts such rather lowbrow visual practices with visualizations used as tools of analytic inquiry.

^3^While being interested in universal semiotic properties, Kress and van Leeuwen emphasize that the use and meaning of images are always culturally and socially situated (Kress and Leeuwen, 2021, p. 3).

^4^This accurate mapping is what lets Stöckl emphasize “iconic” semiotic features of images. Iconicity means representing objects/structures by resemblance, which refers to the icon as a sign category introduced by Peirce (1960). Freyberg (2019) examined iconic properties of visualizations in DH research.

^5^The documentation used as data for the visualizations is taken from two editions of Eyre rolls: The data analyzed for the visualizations in Figures 1, 2 represents the court rolls from the Berkshire Eyre in 1248, edited by Clanchy (1965). The data for the visualization in Figure 3 is taken from the edition of the Huntingdonshire Eyre court rolls from 1286 (DeWindt, 1981).

^6^The printed editions were digitized by Silke Schwandt following the digitization workflow described in Jentsch and Porada (2020).

^7^We used the SpaCy model “Latin [0.2.0],” available via nopaque.uni-bielefeld.de, to lemmatize the word forms.

^8^The visualization technique follows Brezina (2018).

^9^Some of the collocates are conjunctions structuring the court documents, like *ideo* and *quod*, some are personal pronouns referring to the people affected by court practices, like *suum* and *eos*. *Misericordia* and *dotem* refer to the outcome of the cases as these terms denote fines and other fees that were paid in compensation or as a “gift” (*dos*) to arrive at a settlement that was acceptable for all parties (*misericordia*=amicable arrangement). *Latrocinium* is larceny and therefore denotes one of the most frequent crimes or the felonies named in the corpus. *Recognitura* are the things that will be recognized by the court; *optulit* means bring something against someone; *malecreditur* means someone has a bad reputation and cannot be trusted.

^10^*Attorna*^*^ has a total frequency in the corpus of 91; *veni*^*^ has a total frequency of 826 in the Berkshire Rolls.

^11^For the social meaning of medieval names see Ingrand-Varenne et al. (2023).

^12^The exact figures on which this percentage is based relate to the total number of cases heard in Huntingdonshire between 1286 and 1288 before the Eyre and the Assize Courts. However, the ratio is the same as for the Huntingdonshire Eyre in 1286. Criminal cases were obviously more often brought to judgment than civil cases.

^13^“Claiming court demanded considerable knowledge of men, tenures, and pleas, together with the ability to state one's case lucidly and persuasively. On that knowledge and ability depended on the maintenance of the lord's jurisdiction, precious not only for its financial perquisites but also because it embodied the lord's power over his men and his right to mediate their quarrels” (Palmer, 1982, p. 119).

^14^This use of visualizations accommodates for what Andreas Fickers plead for as an “Update of hermeneutics” (Fickers, 2020).
